# Annual Report of the City Inspector of the City of New York, for the Year 1853

**Published:** 1854-08

**Authors:** Thomas K. Downing

**Affiliations:** City Inspector


*Annual Report of the City Inspector of the City of New York, for the year* 1853. By THOMAS K. DOWNING, City Inspector.

We extract the general summary for 1853:

| | | |
|---|---|---|
| "Total number of deaths reported for interment, | | 22,702 |
| Deduct number of *still-born*, | 1,575 | |
| " " " Premature births, | 355 | |
| | 1,939 | |
| " " from Malformation, | 77 | |
| " " " Old Age, | 175 | |
| | —— | 2,202 |
| Leaves the number of deaths occurring from casualties and diseases, | | 20,500 |
| Deduction made of the number of deaths from various casualties, suicides, &c., as per table of external causes, | | 1,025 |
| Leaves total number of deaths from disease alone, | | 19,475 |
| The total number of Whites reported is, | 22,225 | |
| " " " " Blacks, " " | 477 | |
| | —— | 22,702 |
| The number of Male Adults is, | 4,473 | |
| " " " " Children is, | 7,757 | |
| Total Males, | —— | 12,230 |
| The number of Female Adults is, | 3,621 | |
| " " " " Children is, | 6,821 | |
| Total Females, | —— | 14,472 |
| The number of Adults is, | 8,124 | |
| " " " Children is, | 14,578 | —— |
| | —— | 22,702" |

The comparative mortality of children and adults will attract notice.

